# Inhibition of NADPH Oxidase by Apocynin Attenuates Progression of Atherosclerosis

**DOI:** 10.3390/ijms140817017

**Published:** 2013-08-19

**Authors:** Kara Kinkade, Jennifer Streeter, Francis J. Miller

**Affiliations:** 1 Department of Internal Medicine, University of Iowa, Iowa City, IA 52242, USA; E-Mails: kara.seaton@hotmail.com (K.K.); jennifer-streeter@uiowa.edu (J.S.); 2 Department of Anatomy and Cell Biology, University of Iowa, Iowa City, IA 52242, USA; 3 Veterans Affairs Medical Center, Iowa City, IA 52242, USA

**Keywords:** NADPH oxidase, apocynin, atherosclerosis, superoxide

## Abstract

Of the multiple sources of reactive oxygen species (ROS) in the blood vessel, NADPH oxidases are the primary source. Whereas several studies have implicated NADPH oxidases in the initiation of atherosclerosis, their roles in disease progression are incompletely understood. Our objective was to determine the potential clinical relevance of inhibiting NADPH oxidase in established atherosclerosis. Using a hypercholesteremic murine model of atherosclerosis (ApoE^−/−^/LDLR^−/−^ (AS) mice on normal chow diet), we first established a time-dependent relationship between superoxide levels and lesion size in AS mice. Next, we identified NADPH oxidase as the primary source of ROS in atherosclerotic lesions. Treatment of aortic segments from AS mice with apocynin, which interferes with NADPH oxidase activation in part by preventing translocation of the subunit p47^phox^, significantly reduced superoxide levels. Moreover, addition of apocynin to the drinking water of AS mice produced a decrease in lesion size as compared to untreated AS mice, with the effect most pronounced in the thoracoabdominal aorta but absent from the aortic arch. Granulocyte function in AS+apocynin mice was suppressed, confirming efficacy of apocynin treatment. We conclude that apocynin attenuates the progression of atherosclerosis in hypercholesterolemic mice, potentially by its ability to inhibit generation of superoxide by NADPH oxidase.

## 1. Introduction

The mechanisms of atherosclerosis are complex, involving initial events of transendothelial migration of inflammatory cells into the vascular wall and formation of fatty streaks and later events that include vascular cell activation and more complex lesion development. The oxidative modification hypothesis implicates reactive oxygen species (ROS) in the pathogenesis of atherosclerosis. As such, lowering of vascular wall ROS should reduce clinical complications associated with atherosclerosis. However, the disappointing results of antioxidant trials in limiting cardiovascular events in humans have highlighted the importance of targeting the enzymatic source of ROS.

Several studies have implicated NADPH oxidase in the development and early progression of atherosclerosis. NADPH oxidase is a primary source of superoxide in the blood vessel, particularly in disease states. NADPH oxidases are multi-subunit complexes that include several Nox homologs that serve as the site of electron transfer from NADPH to molecular oxygen to generate superoxide. Of the seven Nox isoforms, only Nox1, Nox2, Nox4 and Nox5 are expressed in the blood vessel, and they differ in their cell-specific expression, mode of activation, subunit requirements, and function [1]. Activation of Nox1 and Nox2 requires association with several cytosolic proteins, Rac, p47^phox^ (or its homolog NoxO1), and p67^phox^ (or its homolog NoxA1). The expression of several of these subunits is elevated in vessels from humans with coronary atherosclerosis [2–6].

Studies investigating of the role of NADPH oxidases in atherosclerosis have primarily utilized genetic models with deficiency or overexpression of a particular subunit [7–14]. For example, deficiency of p47^phox^ [8,9], Nox1 [11], or Nox2 [10] but not Nox4 [15] attenuates lesion formation in the aorta of hypercholesterolemic mice. However, a limitation of these studies in genetically modified mice is that the mechanisms of the initiation of disease may be distinct from those involved in disease progression. As such, this limits the clinical relevance of the suggested benefit of NADPH oxidase deficiency.

The objective of our study was to determine whether pharmacologic inhibition of NADPH oxidase activity after the development of atherosclerosis either blocked progression or induced regression. In this study, we identify a time-dependent increase in plaque area and ROS levels in the aorta of hypercholesterolemic mice. Treatment with the NADPH oxidase inhibitor apocynin beginning at 17 weeks of age and continued for an additional 17 weeks reduced vascular ROS and attenuated lesion progression.

## 2. Results and Discussion

### 2.1. Association between Lesion Size and Superoxide Levels in Atherosclerotic Mice

To examine the relationship between lesion size and ROS production in atherosclerosis, we performed studies in ApoE^−/−^ LDLR^−/−^ atherosclerotic (AS) mice on normal chow, which have total cholesterol levels of 600–800 mg/dL (Table 1).

**Figure 1:**
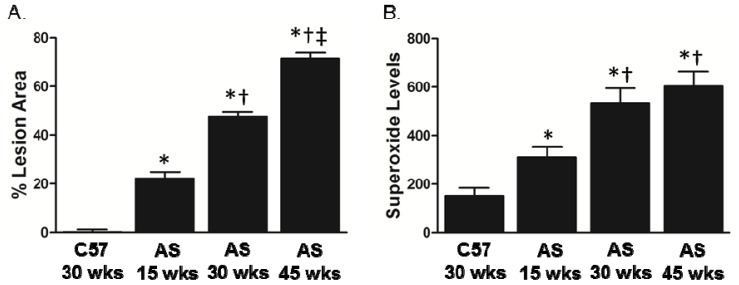
Lesion size continues to progress over time, whereas superoxide levels plateau at 30 weeks in aorta of AS mice. (**A**) Lesion area; (**B**) Superoxide levels (relative light units (RLU)/min/mm^2^) as determined by lucigenin-enhanced chemiluminescence (*n* = 6). * *p* < 0.05 *vs.* C57Bl/6 at 30 weeks; ^†^*p* < 0.05 *vs.* AS at 15 weeks; ^‡^*p* < 0.05 *vs.* AS at 30 weeks by one-way ANOVA with Tukey’s multiple comparison test.

Assessment of lesion area in the aorta demonstrated a time-dependent increase in the AS mice as compared to C57 control mice (Figure 1A). Superoxide levels, as measured by lucigenin-enhanced chemiluminescence, were significantly elevated at 15 weeks of age in AS mice and were further increased at 30 weeks, after which they plateaued (Figure 1B).

### 2.2. Increased Superoxide in AS Aorta Is Derived from NADPH Oxidase

**Figure 2:**
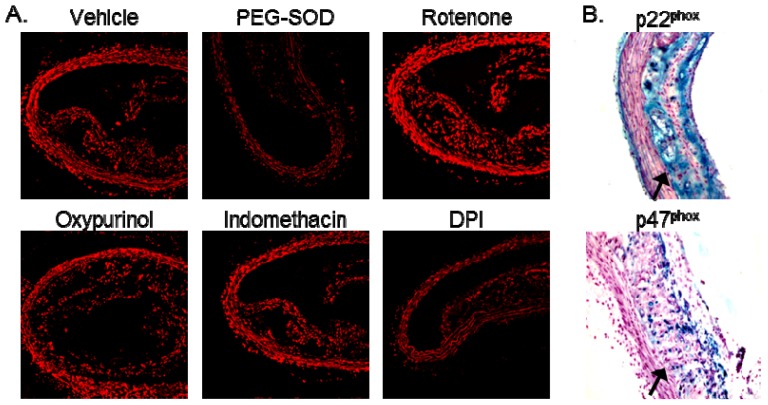
Source of superoxide in AS aorta. (**A**) Micrographs of sequential sections of aorta from AS mice were obtained after staining with dihydroethidium (DHE). Tissue sections were pretreated with polyethylene glycol-superoxide dismutase (PEG-SOD) or indicated inhibitors prior to DHE staining; (**B**) Immunostaining of p22^phox^ and p47^phox^ in AS aorta. Staining with no primary antibody served as a negative control (data not shown).

**Figure 3:**
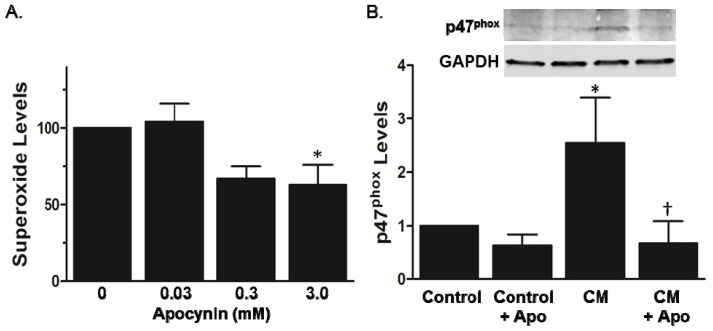
Apocynin blunts superoxide levels and p47^phox^ membrane translocation in AS aorta. (**A**) Superoxide levels (RLU/min/mm^2^) in aortic segments were determined by lucigenin-enhanced chemiluminescence after incubation with indicated concentrations of apocynin. Data are presented relative to vehicle control (*n* = 4). * *p* < 0.05 *vs.* no treatment by one-way ANOVA with Dunnett’s multiple comparison test; (**B**) p47^phox^ translocation to the membrane was examined by Western blotting in membrane fractions from normal aortic segments treated with cytokine mix (CM) in the absence or presence of apocynin. The cytosolic fraction was blotted with anti-GAPDH. Summary data are normalized to GAPDH and then to Control for each experiment (*n* = 3). * *p* < 0.05 *vs.* Control; ^†^*p* < 0.05 *vs.* CM.

We next determined the enzymatic source of the increased vascular superoxide in AS by incubating aortic segments from AS mice with inhibitors of mitochondria (rotenone), xanthine oxidase (oxpurinol), arachidonic metabolism (indomethacin), and flavo enzymes (dipheneylene iodonium/DPI). As compared to vehicle control, treatment with polyethylene glycol-superoxide dismutase (PEG-SOD) abolished superoxide production as detected by dihydroethidium (DHE) fluorescence (Figure 2A), confirming that the fluorescent signal observed in AS aortic segments is from superoxide. Whereas rotenone, oxypurinol, and indomethacin did not decrease superoxide levels in AS vessels, treatment with DPI produced a marked reduction in DHE fluorescence (Figure 2A). These data suggest a potential role for NADPH oxidase in the generation of superoxide in AS aorta. Expression of the NADPH oxidase subunits p22^phox^ and p47^phox^ was increased in the AS lesions as compared to the adjacent medial layer (Figure 2B), consistent with previous reports in human disease [3,4,16]. In control mice, there is no lesion, precluding staining for p22^phox^ and p47^phox^ in the neointima (data not shown). Moreover, treatment with apocynin significantly decreased superoxide production in the AS aortic segments (Figure 3A). In the setting of NADPH oxidase activation, apocynin decreased recruitment of p47^phox^ to the membrane (Figure 3B), consistent with previous reports in leukocytes [17,18].

### 2.3. Effect of Apocynin on Atherosclerosis in the Aorta

**Figure 4:**
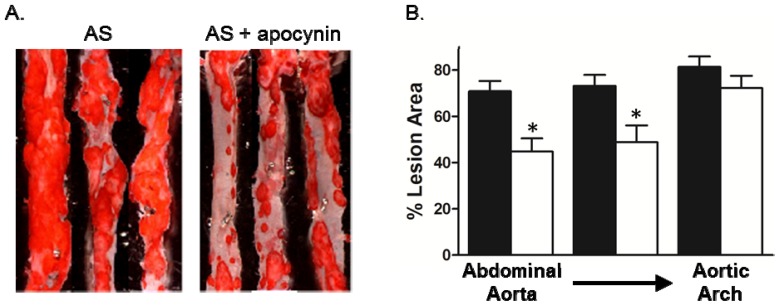
Apocynin inhibited lesion progression in thoracoabdominal aorta but not the aortic arch. Apocynin (500 mg/L) was adding to the drinking water of AS mice at 16–18 weeks of age. Aorta were stained with oil red O at 34–35 weeks of age. (**A**) Representative images of oil red O staining of aorta en face; (**B**) Summary data of lesion area calculated as the percent of total area (*n* = 10–12). Black bars, untreated; white bars, apocynin * *p* < 0.05 *vs.* untreated similar aortic region by one-way ANOVA with Tukey’s multiple comparison test.

We next examined whether treatment with apocynin after the development of atherosclerosis in mice either blocked progression or induced regression by treating mice with apocynin beginning at ~17 weeks of age until ~35 weeks of age. Apocynin had no effect body weight or total cholesterol levels (Table 1). Treatment with apocynin produced a marked reduction in the lesion size in the thoracic and abdominal aorta, with no detectable effect on the aortic arch (Figure 4A,B). The mechanisms involved in lesion formation may differentially activate cellular signaling pathways, including NADPH oxidases. For example, deficiency of Nox1 but not p47^phox^ or Nox2 reduces lesion size in the aortic sinus of hypercholesterolemic mice [8,10,11].

**Figure 5:**
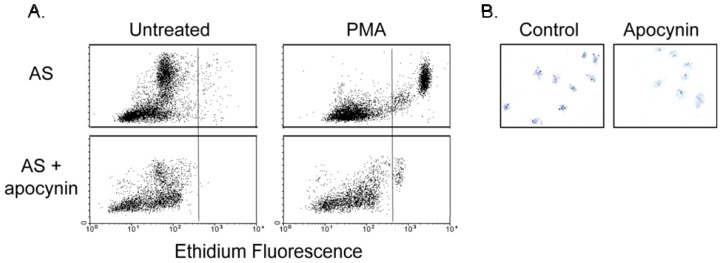
Apocynin decreases respiratory burst in blood leukocytes, indicative of decreased NADPH oxidase activity. (**A**) Flow cytometric analysis of ethidium fluorescence of blood leukocytes collected from control and apocynin-treated mice. 2-Hydroxyethidium fluorescence represents superoxide activation of DHE; (**B**) Nitroblue tetrazolium (NBT) staining of neutrophils after LPS activation. Data are representative of 3–4 experiments.

To confirm that animals treated with apocynin had decreased NADPH oxidase function *in vivo*, we examined the respiratory burst activity of blood leukocytes. There was marked reduction in respiratory burst activity of leukocytes collected from apocynin treated mice with only 475 ± 229 leukocytes increasing superoxide generation in response to PMA compared to 2419 ± 420 leukocytes in control mice (Figure 5A). Similar results were obtained using DHE and dihydrorhodamine (DHR) for detection of superoxide (data not shown). Nitroblue tetrazolium (NBT) assay with LPS activation confirmed decreased NADPH oxidase activity of neutrophils from apocynin treated mice (Figure 5B).

In summary, our data demonstrate an age-dependent increase in lesion size and ROS until later stages of disease, where lesions continue to develop without an associated increase in superoxide levels. The increased superoxide is derived from NADPH oxidases. Inhibition of NADPH oxidase with apocynin reduces vascular ROS and attenuates lesion progression, particularly in the descending aorta.

Evidence from experimental animal and human studies indicate increased generation of ROS in atherosclerosis, which is the cumulative effect of increased superoxide generation in multiple cell types within the diseased vessel wall [19–21]. Accordingly, we detected increased superoxide levels not only within the atherosclerotic lesion but throughout the aorta from hypercholesterolemic mice. Numerous studies have implicated NADPH oxidases as the primary source of superoxide in vascular disease [22,23]. The expression patterns of the multiple NADPH oxidase catalytic (Nox) subunits differ between cell types, resulting in different contributions to disease development. For example, in ApoE^−/−^ mice, whereas the deficiency of Nox2 reduces lesion size in the aorta by ~45%, deficiency of Nox1 reduces lesion area by ~30% [10,11]. This demonstration of a greater role for Nox2 in plaque development reflects the contribution of Nox2-containing inflammatory cells to atherogenesis. Indeed, a major limitation of the Nox2 knockout studies is the inability to differentiate the contribution of Nox2 in vascular cells from that of inflammatory cells. Similarly, ApoE null mice that are deficient in p47^phox^, a regulatory subunit for both Nox1 and Nox2, have a greater reduction in lesion size than mice deficient in either Nox1 or Nox2 alone [8,10,11]. However, it is important to note that these studies utilized genetic deficiency of NADPH oxidase subunits, which will impact all stages of atherosclerosis. As a complex disease, the processes involved in the initiation of atherosclerosis are likely to be distinct from those in the more advanced lesion. An advantage of our study is that it has clinical relevance given that intervention would occur after the development of disease.

Apocynin is a naturally-occurring methoxy-substituted catechol. The activity of apocynin is dependent on its oxidation and subsequent formation of the symmetrical dimer, di-apocynin [21,24]. The metabolically-active compound of apocynin inhibits the intracellular assembly of NADPH oxidases [17,24]. Others have shown that apocynin inhibits p47^phox^ translocation to the membrane in monocytes [18], and our data in Figure 3 provide the first evidence, to the best of our knowledge, that apocynin also prevents translocation of p47^phox^ in vascular cells. As such, apocynin can inhibit activation of multiple Nox subunits that complex with p47^phox^ in a wide range of cell types. Furthermore, there is indirect evidence that apocynin inhibits NoxO1 [25], the p47^phox^ homolog that is also involved in activation of Nox1 [26]. We found that apocynin inhibited superoxide production in aorta and leukocytes, suggesting that the protective effects of apocynin are due to inhibition of both Nox1- and Nox2-derived ROS. Our study does not allow us to differentiate the relative effects of apocynin in vascular cells *vs.* leukocytes, but, using a combination approach of p47^phox−/−^ mice and bone marrow transplantation in AS mice, another study demonstrated a role for p47^phox^ in both the vascular wall and leukocytes [9].

A limitation of this study is that apocynin has been criticized as an inhibitor of NADPH oxidases. In vascular cells, apocynin has antioxidant properties independent of inhibiting NADPH oxidase [21]. However, the antioxidant effect of apocynin is predominant in the absence of myeloperoxidase (MPO), necessary for the formation of active dimers. In contrast to cultured vascular cells, MPO and other peroxidases are present within the disease vessel wall [27] and in macrophages [28] from AS mice. Furthermore, apocynin has been shown to be pro-oxidant [29]. Despite these potential limitations, our data demonstrated that treatment of AS mice with apocynin reduced progression of atherosclerosis. Our results do not distinguish between the contribution of the vascular wall and inflammatory cells to the progression of disease. Indeed, our data with respiratory bursts in leukocytes suggest that the effect of apocynin on lesion size may be solely through inhibition of the inflammatory mediators of atherosclerosis. This observation is consistent with a recent study [30] and has potential clinical significance since apocynin and other general inhibitors of NADPH oxidase may create a chronic granulomatous-like disease. Nonetheless, there is increased recognition of various drugs, including statins and angiotensin-converting enzyme inhibitors, and the development of small molecule inhibitors that target the activation of NADPH oxidases to attenuate the progression of vascular disease [31].

## 3. Experimental Section

### 3.1. Animals and Tissue Preparation

All experiments were performed within the guidelines of the National Institute of Health Guide for the Care and Use of Laboratory Animals, and the protocols approved by the Animal Care and Use Committee at the University of Iowa. The atherosclerotic mice used in this study were apolipoprotein E and low density lipoprotein receptor deficient mice (ApoE^−/−^/LDLR^−/−^) of either sex and ranging in age from 15 to 45 weeks of age. C57BL/6 mice (Jackson Laboratories) were used as controls and were studied at 30 weeks of age. All mice were maintained with normal diet (6% fat, Purina) throughout the study. In some studies, apocynin (3 mM) was added to the drinking water of mice starting at ~17 weeks of age and were maintained on apocynin for a duration of ~17 weeks. Age matched AS mice maintained on regular drinking water were used as controls. Mice were sacrificed between the ages of 15 and 45 weeks by injection of pentobarbital (IP, 150 mg/kg). The aortic arch, thoracic and abdominal aortae were removed, placed in Krebs buffer (4 °C) and dissected free of loosely adhering tissue. Arches were then incubated in phenol red free Dulbecco’s Modified Eagle’s Medium (DMEM) for approximately 1 h at 36 °C before performing lucigenin-enhanced chemiluminescence as described below. After chemiluminescence assays, arches were prepared for lesion size assessment by oil red O staining. In other experiments, aortae were processed for membrane fraction as described below.

### 3.2. Total Cholesterol Measurements

At the time of sacrifice, 1.0 mL of blood was collected from each mouse via cardiac ventricular puncture and placed in heparinized tubes for plasma evaluation (cholesterol) by enzymatic assay (Boehringer Mannheim).

### 3.3. Detection of ROS

#### 3.3.1. DHE Staining

Aortic segments (4 mm) were rinsed in cold PBS and flash-frozen in OCT as previously described [20]. Sections (30 μm) were incubated for 30 min in DHE (10 μM), and examined by fluorescent confocal microscopy (excitation at 488 nm and detection using a 585 nm long-pass filter). Where indicated, sections were pretreated with PEG-SOD (250 U/mL), mitochondrial inhibitor rotenone (0.1 mM), xanthine oxidase inhibitor oxypurinol (0.1 mM), arachidonate metabolism inhibitor indomethacin 10 μM), or flavoenzyme inhibitor DPI (0.1 mM) prior to addition of DHE.

#### 3.3.2. Lucigenin-Enhanced Chemiluminescence

Superoxide production was measured in aortic arches by lucigenin-enhanced chemiluminescence. Vessel segments were incubated in phenol red free DMEM for approximately 1 h at 36 °C. In some experiments, segments were treated with apocynin (0.03 mM, 0.3 mM, 3 mM). Segments were then placed in cuvettes with 0.5 mL phosphate buffered saline (PBS) and lucigenin (25 μM). After a 1.5 min delay time, measurements were taken every 30 seconds for 5 min on a Monolight luminometer. Measurements were reported as emitted light in relative light units (RLU) and were averaged across the 5-minute reading time. Data were normalized to the surface area for each vascular segment, which was obtained with NIH Image J.

### 3.4. p47^phox^ Membrane Translocation

Aortic segments were incubated in phenol red free DMEM for 1 h at 37 °C, followed by treatment with apocynin (1 mM) for 60 min, then cytokine mix (tumor necrosis factor-α, 10 ng/mL + lipopolysaccharide, 10 μg/mL) to activate NADPH oxidase for an additional 10 min. Aortae were flash frozen in liquid nitrogen, homogenized, sonicated, and centrifuged to remove debris. Supernatants were then centrifuged at 100,000× *g* for 1 h at 4 °C. The pellet was resuspended in RIPA buffer supplemented with protease inhibitor cocktail, followed by Western blotting with anti-p47^phox^ (rabbit anti-sera as a gift from Dr. William Nauseef, University of Iowa). The supernatant was blotted with anti-GAPDH.

### 3.5. Lesion Assessment

The staining of lipids was performed by oil red O. Following chemiluminescence measurements, the aortic arch, thoracic and abdominal aortae were cut longitudinally and pinned to lay flat. Tissue was then fixed in 7% formaldehyde, rinsed in 60% isopropanol for 30 seconds, and stained in oil red O for 10 min. Tissue was then rinsed in 60% isopropanol briefly and then washed in running water for 2–3 min. Hematoxylin was used as a counter stain. NIH Image J was used to assess the area of the atherosclerotic lesion. Lesion size was calculated as a ratio of oil red O positive aortic tissue to total surface area.

### 3.6. Immunohistochemistry

Aortic sections (8 μm thick) were washed with PBS and treated first with normal horse serum, and then with p47^phox^ (1:100) and p22^phox^ (1:100) polyclonal antibodies and appropriate secondary antibodies as previously described [32]. Sections were visualized by light microscopy.

### 3.7. Respiratory Burst Measurement

#### 3.7.1. Flow Cytometry

Leukocyte respiratory burst activity was examined as previously described [33]. Approximately 1 mL blood was collected by cardiac puncture in a sodium citrate containing tube. Contaminating erythrocytes were lysed by adding 20 mL of lysis buffer (VitaLyse ByoErgonomicks). After 45 min at room temperature, cells were pelleted by centrifugation for 5 min at 4 °C and washed twice in cold Hanks buffered saline solution (HBSS) and re-suspended in cold HBSS. Cells were incubated with DHE (5 μM) for 10 min, and then phorbol myristate acetate (PMA, 1 μM) was added for 30 min at 37 °C to activate neutrophils. The samples were placed on ice and analyzed using a FACSort flow cytometer (Becton Dickinson) and CellQuest software. Leukocytes were identified on the basis of forward angle and side scatter light signals and gated appropriately. The time elapsed from withdrawal of blood samples until data acquisition was less than 3 h.

#### 3.7.2. NBT Assay

Granulocyte function in mice was assessed in a nitroblue tetrazolium (NBT) slide test in a modified protocol as previously described [34]. In the presence of superoxide, NBT is reduced to blue formazan. Coverslips were incubated in 0.05 mg lipopolysaccharide B per 100 mL of distilled water overnight and then dried. A drop of whole blood, obtained by cardiac puncture at the time of harvesting aorta, was placed on the endotoxin coated coverslip and incubated at 37° for 45 min in a humidified chamber. The coverslip was then washed with saline then treated with a solution containing serum and NBT (1.68 mg NBT dissolved in 0.9 mL PBS and mixed with 0.5 mL mouse serum). The coverslip was incubated for 20 min at 37° in a humidified chamber, then rinsed, air dried and fixed in methanol. The slide was counterstained with nuclear fast red and viewed with a light microscope.

### 3.8. Statistical Analysis

Data are presented as mean ± standard error. Statistical significance was assessed by one-way ANOVA with Tukey’s or Dunnett’s post-test analysis. A *p* value < 0.05 was considered statistically significant.

## 4. Conclusions

In conclusion, our data add to the existing literature, which has primarily relied on genetically modified mice, by demonstrating that a pharmacologic inhibitor of NADPH oxidase can attenuate atherosclerosis. Furthermore, our data extend the role of NADPH oxidases in atherogenesis by revealing that inhibiting NADPH oxidase after disease development is sufficient to limit progression.

## Figures and Tables

**Table 1:** Weight and serum cholesterol levels. Values are mean ± SE (*n* = 3–12).

	Body weight *(*grams)	Cholesterol (mg/dL)
C57BL/6	30 ± 1	91 ± 5
AS (15 weeks)	31 ± 2	749 ± 148
AS (30–35 weeks)	28 ± 1	596 ± 52
AS + apocynin (34–35 weeks)	28 ± 1	592 ± 155
